# A Population-Based Study of Gastroesophageal Reflux Disease and Sleep Problems in Elderly Twins

**DOI:** 10.1371/journal.pone.0048602

**Published:** 2012-10-31

**Authors:** Anna Lindam, Catarina Jansson, Helena Nordenstedt, Nancy L. Pedersen, Jesper Lagergren

**Affiliations:** 1 Unit of Upper Gastrointestinal Research, Department of Molecular Medicine and Surgery, Karolinska Institutet, Stockholm, Sweden; 2 Division of Insurance Medicine, Department of Clinical Neuroscience, Karolinska Institutet, Stockholm, Sweden; 3 Department of Medical Epidemiology and Biostatistics, Karolinska Institutet, Stockholm, Sweden; 4 Division of Cancer Studies, Kinǵs College London, London, United Kingdom; University of Hong Kong, Hong Kong

## Abstract

**Background & Aims:**

Previous studies indicate an association between sleep problems and gastroesophageal reflux disease (GERD). Although both these conditions separately have moderate heritabilities, confounding by genetic factors has not previously been taken into account. This study aimed to reveal the association between sleep problems and GERD, while adjusting for heredity and other potential confounding factors.

**Methods:**

This cross-sectional population-based study included all 8,014 same-sexed twins of at least 65 years of age and born in Sweden between 1886 and 1958, who participated in telephone interviews in 1998–2002. Three logistic regression models were used 1) external control analysis, 2) within-pair co-twin analysis with dizygotic (DZ) twin pairs discordant for GERD, and 3) within-pair co-twin analysis with monozygotic (MZ) twin pairs discordant for GERD. Odds ratios (ORs) with 95% confidence intervals (CIs) were calculated and adjusted for established risk factors for GERD, i.e. sex, age, body mass index (BMI), tobacco smoking, and educational level.

**Results:**

A dose-response association was identified between increasing levels of sleep problems and GERD in the external control analysis. Individuals who often experienced sleep problems had a two-fold increased occurrence of GERD compared to those who seldom had sleep problems (OR 2.0, 95% CI 1.8–2.4). The corresponding association was of similar strength in the co-twin analysis including 356 DZ pairs (OR 2.2, 95% CI 1.6–3.4), and in the co-twin analysis including 210 MZ pairs (OR 1.5, 95% CI 0.9–2.7).

**Conclusion:**

A dose-dependent association between sleep problems and GERD remains after taking heredity and other known risk factors for GERD into account.

## Introduction

Gastroesophageal reflux disease (GERD), defined as recurrent regurgitation of stomach contents into the esophagus which causes troublesome symptoms or complications [1], is a public health problem in the Western world, affecting up to 20% of adult populations [2], [3]. GERD is associated with a negative effect on health-related quality of life [4], and an increased risk of esophageal adenocarcinoma [5]. Established risk factors for GERD are heredity, obesity, and tobacco smoking [2], [6], [7]. Twin studies have shown that 31–43% of the variation in liability to GERD could be explained by genetic factors [8], [9], and the gene collagen type III alpha 1 has been associated with GERD and hiatus hernia [10]. Sleep problems are another common health concern, affecting about one-third of the adult population in industrialized countries [11]. Genetic factors account for 33–44% of the variance in sleep quality and sleep disturbance [12], [13]. Obesity is also a known risk factor for sleep problems, both independently and through contributing to diabetes and the metabolic syndrome. This syndrome increases the risk of cardiovascular disease, which in turn, as well as diabetes alone, is linked with sleep problems [14]. Previous research indicates that sleep problems are associated with GERD [15], [16], but although both these conditions are associated with a moderate heritability, confounding by genetic factors has not been adjusted for in existing studies. To address the association between sleep problems and GERD, and for the first time, take genetic and early environmental factors into account, a large population-based twin study was conducted.

**Table 1:** Characteristics of 8,014 twin study participants with and without gastroesophageal reflux disease (GERD).

		GERD; n (%)	Not GERD; n (%)	P-value^a^
**All participants**		1,327 (17)	6,687 (83)	
**Sex**	Men	544 (41)	2,868 (43)	
	Women	783 (59)	3,819 (57)	0.2023
**Age (years)**	65–74	831 (63)	4,014 (60)	
	≥75	496 (38)	2,673 (40)	0.0773
**Education (years)**	≤9	923 (70)	4,666 (70)	
	10–12	239 (18)	1,168 (18)	
	>12	159 (12)	813 (12)	0.2200
	Missing	6 (0)	40 (1)	
**Body mass index**	<25 (normal)	572 (43)	3,488 (52)	
	25–30 (overweight)	563 (42)	2316 (35)	
	>30 (obese)	130 (9)	459 (7)	<.0001
	Missing	62 (5)	424 (6)	
**Tobacco smoking**	Current smokers	138 (10)	764 (11)	
	Previous smokers	473 (36)	2,040 (31)	
	Never smokers	654 (49)	3,594 (54)	0.0007
	Missing	62 (5)	289 (4)	

a Pearson’s chi-square test estimating the associations between GERD and sex, age, education, body mass index and tobacco smoking separately.

## Methods

### Study Design

This nationwide population-based study was based on the Swedish Twin Registry, described in detail elsewhere [17]. Briefly, this register was established in the late 1950s and several data collections have been undertaken since then. In the present study, data from the Screening Across the Lifespan Twin Study (SALT) were used. All twins born in Sweden before 1958 and alive at the time of the data collection were invited to participate. In total, 45,809 twins (74% response rate) completed structured computer-assisted telephone interviews during March 1998 through November 2002. In this study, only twins who were at least 65 years old at the time of the interview were included, since sleep problem items were assessed in this age group only. Two cross-sectional study designs were used. First, all participating twins were included in a so-called external analysis. Second, same-sexed dizygotic (DZ) and monozygotic (MZ) pairs discordant for GERD were included separately, using a nested case-control design to assess the influence of heredity and early environmental factors. The participants received written information about the study by mail 10 days before the interviews and verbal informed consent was obtained from all participants at the time of the telephone interviews. As the participants were interviewed by phone and not in person only verbal consent could be obtained. Following the interviews, a letter was sent to all participants confirming that they had consented to participate in the study. Information concerning who to contact if they wished to withdraw their consent was also provided. The consent was recorded in the computerized system used for recording the answers during the interview. The study, including the consent procedure, was approved by the Regional Ethical Review Board in Stockholm, Sweden (Dnr: 97-051, 00-132 and 2010/582-31/1).

**Table 2:** The distribution of gastroesophageal reflux disease (GERD) in same-sexed dizygotic (DZ) and monozygotic (MZ) twin pairs.

		DZ pairs; n (%)	MZ pairs; n (%)
**Concordant, both twins have GERD**		51 (4)	52 (6)
**Concordant, neither twin has GERD**		1004 (71)	647 (71)
**Discordant for GERD** a		356 (25)	210 (23)
**Sex, same-sexed discordant pairs**	Men	137 (38)	80 (38)
	Women	231 (61)	130 (62)
**Age (in years), discordant pairs**	64–74	259 (73)	149 (71)
	≥75	97 (27)	61 (29)

a One twin has GERD, and the other not.

### Assessment and Definition of Gastroesophageal Reflux Disease (GERD)

GERD was assessed by 10 validated questions [7], [8]. During the telephone interviews, all participants were asked if they had heartburn, pain behind the breastbone, or regurgitation of bitter fluid or acidic fluids into the mouth. If a positive response was given to any of these questions, seven additional questions were asked regarding duration and frequency of symptoms, radiation of pain towards the neck, antacid relief, and use of histamine-receptor antagonists or proton pump inhibitors. GERD was defined by at least weekly occurrence of pain behind the breast bone, regurgitation of bitter or acidic fluids, or heartburn. Participants who reported having pain behind the breast bone 1–3 times a month in combination with i) waking up at night due to the pain, ii) use of medications to prevent the pain, iii) pain radiating towards the neck, or iv) antacids not reducing the pain, were also classified as having GERD. The definition of GERD used in this study is consistent with the Montreal definition [1].

**Table 3:** Associations between sleep problems and the occurrence of gastroesophageal reflux disease (GERD) among 7,857 twins.

			External analysis			
		GERD^a^	Crude		Adjusted^b^	
**Exposure**		n (%)	OR	95% CI	OR	95% CI
**Insomnia index** c	Seldom	456 (35)	1.0	(reference)	1.0	(reference)
	Sometimes	438 (34)	1.5	1.3–1.7	1.5	1.3–1.7
	Often	403 (31)	2.1	1.8–2.4	2.0	1.8–2.4
**Not rested when waking up**	Seldom	842 (65)	1.0	(reference)	1.0	(reference)
	Sometimes	261 (20)	1.5	1.3–1.8	1.5	1.3–1.8
	Often	197 (15)	1.8	1.5–2.1	1.7	1.5–2.1
**Disturbed sleep**	Seldom	872(67)	1.0	(reference)	1.0	(reference)
	Sometimes	269 (21)	1.6	1.4–1.9	1.7	1.4–1.9
	Often	162 (12)	2.0	1.7–2.5	2.0	1.6–2.4
**Waking up too early**	Seldom	709 (54)	1.0	(reference)	1.0	(reference)
	Sometimes	357 (27)	1.3	1.1–1.5	1.3	1.1–1.5
	Often	240 (18)	1.9	1.6–2.3	1.9	1.6–2.3

OR, odds ratio; CI, confidence interval.

a The different numbers of GERD cases for the four sleep exposures are due to different numbers of missing observations in the sleep questions.

b ORs adjusted for age, sex, educational level, body mass index, and tobacco smoking.

c The insomnia index was constructed by combining the three different sleep problem questions and 0 points were given for “seldom”, 1 point for “sometimes” and 2 points for “often”. The highest scores, 4–6 points or if the participant had answered “often”, were then classified as “often having sleep problems”, 1–3 points as “sometimes having sleep problems” and 0 as ”seldom having sleep problems”.

### Assessment and Definition of Sleep Problems

Sleep problems were assessed in the SALT interview by the Karolinska Sleep Questionnaire [18], [19]. This questionnaire included three items assessing how often during the last six months the participants had any of the following problems: “not rested when waking up”, “disturbed sleep” and “waking up too early and not able to go back to sleep”. Each item had five response alternatives: “never”, “seldom”, “sometimes”, “mostly” or “always”. These responses were categorized a priori into three groups: i) “seldom” (including “never” and “seldom”), ii) “sometimes”, or iii) “often” (including “mostly” and “always”). In addition, an insomnia index was constructed based on the responses to the three items by giving 0 points for the response alternative “seldom”, 1 point for “sometimes”, and 2 points for “often”. A score of 4–6 points or any item with a response of “often” were classified as “often having sleep problems”, 1–3 points was defined as “sometimes having sleep-problems”, and 0 points represented “seldom having sleep problems”.

**Table 4:** Associations between sleep problems and the occurrence of gastroesophageal reflux disease (GERD), among 365 dyzygotic (DZ) twin pairs discordant for GERD.

		Co-twin within-pair analysis DZ twins			
		Crude		Adjusted^a^	
**Exposure**		OR	95% CI	OR	95% CI
**Insomnia index** b	Seldom	1.0	(reference)	1.0	(reference)
	Sometimes	1.6	1.1–2.3	1.5	1.0–2.2
	Often	2.2	1.5–3.4	2.2	1.5–3.4
**Not rested when waking up**	Seldom	1.0	(reference)	1.0	(reference)
	Sometimes	1.6	1.0–2.3	1.5	1.0–2.3
	Often	2.0	1.3–3.2	2.1	1.3–3.3
**Disturbed sleep**	Seldom	1.0	(reference)	1.0	(reference)
	Sometimes	1.8	1.2–2.8	1.9	1.2–2.9
	Often	1.8	1.1–2.9	1.7	1.0–2.8
**Waking up too early**	Seldom	1.0	(reference)	1.0	(reference)
	Sometimes	1.2	0.8–1.8	1.1	0.7–1.6
	Often	1.8	1.1–2.9	1.7	1.1–2.7

OR, Odds ratio; CI, confidence intervals.

*^a^*ORs adjusted for educational level, body mass index, and tobacco smoking. Genetic and early environmental factors, sex and age are adjusted for by the within pair structure.

*^b^*The insomnia index was constructed by combining the three different sleep problem questions and 0 points were given for “seldom”, 1 point for “sometimes” and 2 points for “often”. The highest scores, 4–6 points or if the participant had answered “often”, were then classified as “often having sleep problems”, 1–3 points as “sometimes having sleep problems” and 0 as ”seldom having sleep problems”.

### Assessment and Definition of Zygosity

Zygosity of the same-sexed pairs was assessed by asking each twin independently the question: “During childhood, were you and your twin partner as alike as ‘two peas in a pod’ or not more alike than siblings in general?” If both twins in the pair answered that they were “alike as two peas in a pod” they were classified as monozygotic (MZ), and if both answered that they “were not more alike than siblings” they were classified as dizygotic (DZ). If the twins answered differently they were categorized as “not determined”. This method of determining zygosity has been shown to be 98% accurate compared to DNA-testing [17].

**Table 5:** Associations between sleep problems and the occurrence of gastroesophageal reflux disease (GERD) among 210 monozygotic (MZ) twin pairs discordant for GERD.

		Co-twin within-pair analysis MZ twins			
		Crude		Adjusted^a^	
**Exposure**		OR	95% CI	OR	95% CI
**Insomnia index** b	Seldom	1.0	(reference)	1.0	(reference)
	Sometimes	1.4	0.9–2.2	1.3	0.8–2.2
	Often	1.6	0.9–2.8	1.5	0.9–2.7
**Not rested when waking up**	Seldom	1.0	(reference)	1.0	(reference)
	Sometimes	1.6	0.9–2.7	1.6	0.9–2.8
	Often	1.9	1.0–3.8	1.9	0.9–3.9
**Disturbed sleep**	Seldom	1.0	(reference)	1.0	(reference)
	Sometimes	1.8	1.0–3.4	1.9	1.0–3.6
	Often	1.5	0.7–3.2	1.4	0.7–3.1
**Waking up too early**	Seldom	1.0	(reference)	1.0	(reference)
	Sometimes	1.5	0.9–2.3	1.5	0.9–2.4
	Often	1.5	0.8–2.8	1.5	0.8–2.9

OR, odds ratio; CI, confidence intervals.

*^a^*ORs adjusted for educational level, body mass index, and tobacco smoking. Genetic and early environmental factors, sex and age are adjusted for by the within pair structure.

*^b^*The insomnia index was constructed by combining the three different sleep problem questions and 0 points were given for “seldom”, 1 point for “sometimes” and 2 points for “often”. The highest scores, 4–6 points or if the participant had answered “often”, were then classified as “often having sleep problems”, 1–3 points as “sometimes having sleep problems” and 0 as ”seldom having sleep problems”.

### Assessment and Definition of Potential Confounders

The potential confounding factors heredity, sex, age, educational level, body mass index (BMI), and tobacco smoking were predefined and selected as they are established risk factors for GERD [2], [7] and also known to be associated with sleep problems [11]. Information regarding sex and age were derived from the personal identity number, a unique 10-digit number assigned to all Swedish residents, including information about sex and birth date. Age at interview was categorized into 65–74 years or ≥75 years. Formal education was assessed in the SALT interview, and grouped into three categories: “0–9 years”, “10–12 years”, or “>12 years”. Self-reported height and weight were used to calculate BMI (weight in kilograms divided by the square of the height [kg/m^2^]), and categorized into three groups in accordance with WHO definitions: “low or normal” (<25), “overweight” (25–30), or “obese” (>30). Finally, tobacco smoking status was assessed in the SALT interview, and the participants were categorized into three groups: “never smokers”, “former smokers”, or “current smokers”. Moreover, as the number of missing observations for BMI, tobacco smoking, and educational level were not negligible (4–11%), missing categories were used for these three variables to retain statistical power in the analyses.

**Table 6:** Intraclass correlations for gastroesophageal reflux disease (GERD) and sleep problems separately.

	Dyzygotic pairs, N = 1 411		Monozygotic pairs, N = 909	
	r^a^	ASE^b^	r^a^	ASE^b^
**GERD**	0.151	0.041	0.359	0.044
**Insomnia index** c	0.197	0.024	0.239	0.029
**Not rested when waking up**	0.069	0.031	0.086	0.039
**Disturbed sleep**	0.207	0.031	0.311	0.038
**Waking up too early**	0.193	0.027	0.308	0.030

a Polychoric correlation, tetrachoric for GERD as GERD only has two categories.

b Asymptotic error of the polychoric/tetrachoric correlations.

*^c^*The insomnia index was constructed by combining the three different sleep problem questions and 0 points were given for “seldom”, 1 point for “sometimes” and 2 points for “often”. The highest scores, 4–6 points or if the participant had answered “often”, were then classified as “often having sleep problems”, 1–3 points as “sometimes having sleep problems” and 0 as ”seldom having sleep problems”.

### Statistical Analyses

The main analyses were performed in three steps. First, external control analyses using unconditional logistic regression were performed. As many twins had a co-twin in the cohort, correction for within-pair dependency was conducted with generalized estimated equations (GEE) to avoid underestimation of the variance. Crude odds ratios (ORs) and ORs adjusted for sex, age, educational level, BMI, and tobacco smoking were estimated. Second, within-pair co-twin analyses with DZ twin pairs were performed using conditional logistic regression. Only complete twin pairs were included in the analyses. Third, within-pair co-twin analyses with complete MZ twin pairs were performed using conditional logistic regression. Crude and adjusted ORs were also calculated for all within-pair analyses. In all three analyses, 95% confidence intervals (CIs) were estimated. By including only twin pairs discordant for GERD, i.e. where one twin had GERD and the other not, it was possible to adjust for genetic and early environmental factors in the within-pair analyses. This is due to the fact that MZ twins share 100% of their genes and DZ twins share on average 50% of their genes, and the large majority of twins share childhood environment. There can be variation in the genome for monozygotic twins too, due to epigenetic changes, and greater variations are found among older twins [20]. An attenuation of the OR from the first step to the second and third indicates that familial and genetic factors, respectively, may confound any association found in the first analysis. In another series of analyses, we assessed the importance of genetic factors for GERD and sleep problems, respectively, and their association by computing intraclass correlations and cross-trait correlations for MZ and DZ pairs. Finally, to investigate whether individuals with nocturnal reflux or individuals who used reflux medications more often suffered from sleep problems than those who did not, sub analyses using Pearson’s chi squared test were performed among individuals reporting heartburn or pain behind the breastbone. All analyses were made using SAS software 9.2 (SAS Institute, Cary, NC).

**Table 7:** Twin cross-trait correlations of sleep problems and gastroesophageal reflux disease.

	Gastroesophageal Reflux Disease					
	All pairs, N = 2 341		Dyzygotic pairs, N = 1 411		Monozygotic pairs, N = 909	
	r^a^	ASE^b^	r^a^	ASE^b^	r^a^	ASE^b^
**Insomnia index** c	0.067	0.025	0.077	0.032	0.043	0.039
**Not rested when waking up**	0.033	0.028	0.065	0.036	−0.012	0.045
**Disturbed sleep**	0.061	0.029	0.050	0.037	0.071	0.046
**Waking up too early**	0.059	0.026	0.041	0.034	0.073	0.041

a Polychoric correlation.

b Asymptotic error of the polychoric correlations.

*^c^*The insomnia index was constructed by combining the three different sleep problem questions and 0 points were given for “seldom”, 1 point for “sometimes” and 2 points for “often”. The highest scores, 4–6 points or if the participant had answered “often”, were then classified as “often having sleep problems”, 1–3 points as “sometimes having sleep problems” and 0 as ”seldom having sleep problems”.

**Table 8:** Associations between nocturnal reflux symptoms, use of reflux medications and sleep problems, within a subsample of 786 twins reporting pain behind the breastbone or heartburn.

		Nocturnal reflux symptoms^a^			Medication use to prevent pain^b^		
		yes	no		yes	no	
**Exposure**		n (%)	n (%)	p-value^c^	n (%)	n(%)	p-value^d^
**Insomnia index** e	Seldom	105 (29)	145 (36)		122 (31)	128 (35)	
	Sometimes	126 (35)	127 (32)		122 (32)	125 (34)	
	Often	131 (36)	127 (32)	0.0964	139 (36)	116 (31)	0.3688
**Not rested when waking up**	Seldom	215 (60)	253 (63)		226 (59)	238 (65)	
	Sometimes	79 (22)	80 (20)		82 (21)	73 (20)	
	Often	67 (19)	67 (17)	0.5781	76 (20)	57 (15)	0.2011
**Disturbed sleep**	Seldom	216 (59)	277 (70)		242 (63)	244 (65)	
	Sometimes	88 (24)	80 (20)		88 (23)	79 (21)	
	Often	60 (16)	44 (15)	0.0134	53 (14)	50 (13)	0.7991
**Waking up too early**	Seldom	176 (49)	224 (56)		208 (54)	188 (51)	
	Sometimes	105 (29)	103 (26)		94 (24)	110 (30)	
	Often	75 (23)	75 (19)	0.1279	82 (21)	74 (20)	0.2886

a Nocturnal reflux symptoms was assessed by the question “Have you woken up during the night due to pain behind the breastbone or heartburn?”.

b Use of reflux medications was assessed by the question “ Have you taken any of the following medications to prevent pain behind the breastbone or heartburn “, followed by a list of proton pump inhibitors used in Sweden at the time of the data collection.

c Pearson’s chi-square test measuring the association between nocturnal reflux symptoms and sleep problems.

d Pearson’s chi-square test measuring the association between use of reflux medications to prevent pain and sleep problems.

e The insomnia index was constructed by combining the three different sleep problem questions and 0 points were given for “seldom”, 1 point for “sometimes” and 2 points for “often”. The highest scores, 4–6 points or if the participant had answered “often”, were then classified as “often having sleep problems”, 1–3 points as “sometimes having sleep problems” and 0 as “seldom having sleep problems”.

## Results

### Study Participants

Among 8,951 twins eligible for inclusion in this study, 937 (10%) were excluded due to missing information on GERD. The remaining 8,014 twins, of whom 1,327 (17%) had GERD, were included in the descriptive analyses. After further exclusion of 157 (2%) twins with missing information on insomnia status, 7,857 remained in the main statistical analyses, while 786 individuals reporting heartburn or pain behind the breastbone were included in the sub analyses. The distribution of sex, age and education was similar between twins with and without GERD, whereas high BMI and tobacco smoking were overrepresented among those with GERD (Table 1). Among the DZ and MZ twin pairs, 25% and 23% were discordant for GERD, respectively (Table 2). The age and sex distributions among DZ twin pairs discordant for GERD were similar to that of the MZ twins (Table 2).

### Association between Sleep Problems and GERD

A dose-response association was observed between increasing sleep problems and occurrence of GERD in the external control analysis (Table 3). Individuals who often had sleep problems according to the insomnia index had a two-fold increased occurrence of GERD compared to those who seldom had sleep problems (OR 2.0, 95% CI 1.8–2.4), while individuals who sometimes had sleep problems had a 50% increased occurrence of GERD (OR 1.5, 95% CI 1.3–1.8). This association was similar in the co-twin analysis with discordant DZ twins (Table 4). Compared to those who “seldom” had sleep problems, the adjusted OR of GERD was 2.2 (95% CI 1.5–3.4) for those who “often” had sleep problems and 1.5 (95% CI 1.0–2.2) for those who “sometimes” had sleep problems. The dose-response pattern remained in the co-twin analysis with discordant MZ twins, although was not statistically significant (Table 5). The adjusted OR for GERD was 1.5 (95% CI 0.9–2.7) for those who “often” had sleep problems, and 1.4 (95% CI 0.8–2.2) for those who “sometimes” had sleep problems, compared to those who “seldom” experienced sleep problems.

The sleep problem items were also analyzed separately and the association between “not rested when waking up” and GERD showed a similar dose-response association as the insomnia index, except in the within-pair model with MZ twins where the estimates were higher (Table 3, 4, 5). The association between disturbed sleep and GERD showed a similar dose-dependent pattern in the external analysis as the insomnia index. However, in the within-pair analyses for DZ twins, the occurrence of GERD in those who “sometimes” had disturbed sleep as opposed to “seldom” was slightly higher (OR 1.9, 95% CI 1.2–2.4) than for those who “often” had disturbed sleep (OR 1.7, 95% CI 1.0–2.8) (Table 3, 4, 5). A similar pattern was seen in the corresponding MZ analysis. Individuals who “often” suffered from waking up too early had an almost two-fold increased occurrence of GERD compared to individuals who seldom did so in the external analysis, and individuals who ”sometimes” suffered from waking up too early had a 30% increased occurrence of GERD (Table 3, 4, 5).

The intraclass correlations for GERD were 0.151 in DZ twin pairs and 0.359 in MZ twin pairs (Table 6). For sleep problems the intraclass correlations ranged from 0.069 (Not rested when waking up to 0.207 (Disturbed sleep) in DZ twin pairs and from 0.086 (Not rested when waking up) to 0.311 (Disturbed sleep) in MZ twin pairs, indicating considerably less genetic variation for sleep problems (Table 6).

The cross-trait correlations for sleep problems and GERD were very weak, ranging from 0.033 (Not rested when waking up and GERD) to 0.067 (Insomnia index and GERD) in “all pairs”, 0.041 (Waking up too early and GERD) to 0.077 (Insomnia index and GERD) in DZ pairs and between −0.012 (Not rested when waking up and GERD) to 0.073 (Waking up too early and GERD in MZ twin pairs (table 7). This suggests that genetic and family environmental factors are of little importance, if at all, for the association between GERD and sleep problems.

Finally, among those who reported waking up at night due to pain behind the breastbone or heartburn 16% “often” had disturbed sleep compared to 11% among those who did not and there was a significant association between nocturnal reflux symptoms and disturbed sleep (χ^2^ 8.6; p-value 0.01) (Table 8). No associations were observed between nocturnal reflux symptoms and frequent sleep problems (insomnia index), “not rested when waking up” or waking up too early (Table 8). The distributions of frequency of sleep problems were similar between the individuals who used reflux medications (i.e. proton pump inhibitors) and those who did not (Table 8) and there was no significant association seen between medication use and sleep problems.

## Discussion

This twin study indicates a dose-response association between sleep problems and GERD, which remained after adjustment for genetic and familial environmental factors, as well as for sex, age, educational level, BMI and tobacco smoking.

When the results from the external analyses (including all twins) were compared with the results from the co-twin within-pair analyses for DZ and MZ twins (discordant for GERD), there were minor differences regarding “sometimes” having sleep problems, while the differences were slightly more pronounced for “often” having sleep problems. The association for “often” having sleep problems compared to “no” sleep problems was somewhat stronger among DZ twins compared to that of all twins, while the association was attenuated in the MZ twin analyses. Such a decrease in effect limited to MZ twins indicates genetic influence or limited statistical power, since the sample size for MZ twins was smaller than for the DZ twins. Measures of intrapair similarity (concordances and intraclass correlations) are greater for MZ than DZ pairs for GERD, supporting previous findings in this cohort that genetic factors are of moderate importance for GERD [8]. However, the MZ:DZ differential is generally lower for indicators of sleep problems compared to two other twin studies [12], [13], and in general suggests that familial environmental influences are more important than genetic factors for these measures of sleep problems. The lack of a difference in the cross-trait correlations for MZ and DZ pairs further supports the conclusion that there is no genetic or familial environmental confounding of the association between sleep problems and GERD.

In a previous population-based cross-sectional case-control study from our group, including 65,333 participants in the county of Nord-Trøndelag in Norway, a positive dose-response association between sleeplessness and GERD was found [15]. Another population-based study using the 2006 US National Health and Wellness Survey of 41,319 participants found that participants with GERD had a two-fold increased occurrence of sleep difficulties compared to those without GERD [16]. Thus, the results of these previous studies are well in line with the results of the present study, and this study further adds that the association persists after adjustment for heredity and family environment.

A proposed mechanism for sleep problems causing GERD is that sleep deprivation leads to esophageal hyperalgesia, i.e. patients with GERD are more pain sensitive to their reflux symptoms when sleep deprived [21]. Another potential mechanism is that medications used for sleep disturbances might provoke or aggravate GERD [22]. We observed no association between reflux medications and disturbed sleep. GERD, on the other hand, might cause or worsen sleep disturbances [16], [23]. Nocturnal reflux might lead to uneasy sleep and repeated awakenings, which are common in GERD patients [23]. This was confirmed in the present study where an association between nocturnal reflux symptoms and disturbed sleep was observed. One model suggests a vicious circle, i.e., that sleep disturbances provoke reflux, which in turn worsens sleep problems, which in turn worsen GERD [24].

Strengths of the present study include the population-based design, the extensive data collection based on structured telephone interviews, and the ability to, for the first time, adjust for genetic and early environmental factors in addition to other known risk factors for GERD and sleep problems. Other advantages include the validated assessment of GERD [5], [8] consistent with the Montreal definition [1]. Sleep problems were assessed using frequency of self-reported insomnia symptoms, which is commonly used in epidemiological studies [11]. Limitations of the study include that only twins aged 65 years or older participated. The results might therefore not be generalizable to younger people. However, as the prevalence of both sleep problems and GERD is high in older ages [25], [26], [27], [28], the results of this study should nevertheless be highly relevant. Another weakness is the cross-sectional study design, which prohibits evaluation of the direction of the association between sleep problems and GERD. However, this association is likely to be bidirectional [24].

In conclusion, this large population-based twin study indicates an association between sleep problems and GERD that remains after adjustment for heredity and familial environmental factors, as well as after other known risk factors for GERD and sleep problems.
